# Long Non-Coding RNAs Expression in Breast Cancer: CBR3-AS1 LncRNA as a Sensitive Biomarker

**DOI:** 10.31557/APJCP.2021.22.9.2897

**Published:** 2021-09

**Authors:** Safoora Torkashvand, Ali Basi, Hossein Ajdarkosh, Nasser Rakhshani, Nahid Nafisi, Seyed Javad Mowla, Ayda Moghadas, Mahshid Mohammadipour, Mohammad Hadi Karbalaie Niya

**Affiliations:** 1 *Kasra Hospital, Tehran University of Medical Sciences, Tehran, Iran. *; 2 *Department of Oncology, Firoozgar Hospital, Iran University of Medical Sciences, Tehran, Iran. *; 3 *Gastrointestinal and Liver Diseases Research Center, Iran University of Medical Sciences, Tehran, Iran. *; 4 *Department of Internal Medicine, Iran University of Medical Sciences, Tehran, Iran. *; 5 *Rasool Akram Medical Complex Clinical Research Development Center (RCRDC), Iran university of medical science Tehran, Iran. *; 6 *Department of Molecular Genetics, Faculty of Biological Sciences, Tarbiat Modares University, Tehran, Iran. *; 7 *Blood Transfusion Research Center, High Institute for Research and Education in Transfusion Medicine, Tehran, Iran. *

**Keywords:** lncRNA, breast cancer, CBR3, AS1, RAB6C, AS1, ZEB2, AS1

## Abstract

Background:

Long non-coding RNAs (LncRNAs) are eminent genes in the human genome that interfere with the regulation of many complexities of organisms and control many of the various biological processes. As a result, it is considered that they may play an important role in different cancers. With regard to the high prevalence of breast cancer and the role of lncRNA, the present study aimed at investigating the expression of various lncRNAs.

Method:

Fresh tissues were obtained from operating rooms of Shariati, Khatamolanbia, and Milad Hospitals (Tehran, Iran) by a surgeon. A total of 45 tumor samples and 45 non-tumor samples (from the margin of tumor) were obtained from the same patients. Relative expression evaluation method was used in Real time PCR. Estrogenn receptor (ER), progesterone receptor (PR), and HER2 expression were analyzed using IHC analyses of each cell block.

Results:

Participants included 44 female and 1 male with the mean age ± SD of 50 ± 12.0 years (range: 23-74). A majority of participants (41/45) were Ductal carcinoma type. Our results showed significant expressions for CBR3-AS1 (P-value=0.0139), RAB6C-AS1 (P-value=0.0023), and ZEB2-AS1 (P-value=0.0289) in comparison with the healthy cells. ROC curve analysis for CBR3-AS1 LncRNA revaled sensitivity more than 70%.

Conclusion:

Although CBR3-AS1, RAB6C-AS1, and ZEB2-AS1 lncRNAs were found to have high expressions in the breast cancer cells, only CBR3-AS1 lncRNA has a high chance to be a breast cancer biomarker.

## Introduction

Breast cancer is ranked as the second most common cancer type after lung cancer. It is the most prevalent cancer in women. According to reports, new cases of breast cancer in 2012 were 1.67 million and it accounts for 25% of all cancers worldwide (Momenimovahed and Salehiniya, 2019). Given the importance of this cancer, it is a significant endeavor to identify the factors involved. Heterogeneity of breast cancers classifies them into tumor groups, which have different types of response to the therapeutic strategies (Fumagalli et al., 2020). 

Detection of the networks involved in cancers has an important role in increasing our knowledge about them, but unfortunately most of these networks are unknown. Long non-coding RNAs (lncRNA) refer to larger than 200 nucleotides transcripts (Wang et al., 2019). Nowadays, thanks to advancements in RNA sequencing technology and computational biology, more lncRNAs have been detected. It is likely that there are 410000 lncRNAs in the human genome. Recent findings have shown the role of lncRNAs in cancer development pathways (El-Ashmawy et al., 2020). Also, regulatory roles of lcnRNAs in various processes such as chromatin organization, transcription, and post transcriptional modification have been described by their interaction with RNAs, DNAs, and proteins (or their complex). The mis-expression of these RNAs can increase the chance of tumor growth and metastasis (Liang et al., 2020). GAPLINC is a 924bp long lncRNA which is located on shorter arm of chromosome 18. This lncRNA has a high expression in gastric cancer and is associated with a low level of patient survival (Roohallah et al., 2019). LncRNA BLACAT1 with chromosomal position of 1q32.1 is a biomarker for muscle invasive bladder cancer due to its high expression in these types of cell and is a complementary biomarker for colorectal cancer (CRC)(Han et al., 2020). Accordingly, this new class of RNA may be a potential biomarker for diagnosis, prognosis, and therapeutic purposes in various cancers. Deregulation of some lncRNAs, such as HOTAIR (Collina et al., 2019), XIST (Salama et al., 2020), MALAT1 (Zheng et al., 2019), and H19 (Elias-Rizk et al., 2020) have been identified in breast cancer samples and cell lines.

LncRNA ZEB2-AS1 is a natural antisense transcript related to 5’UTR of zinc finger E-box binding homeobox 2 (ZEB2) with probable role in regulation of ZEB2 expression. According to the ZEB2 role in various cancers, its expression regulation by ZEB2-AS may have an impact on cancer progression (Mahboobeh et al., 2020). On the other hand, due to the role of *RAB6C* gene (coding a centrosomal protein) in cell division (Kubiak et al., 2020), lcnRNA RAB6C-AS1 can be considered as a possible factor in the development of various cancers. Previously, the effect of carbonyl reductase gene (*CBR3-AS1*) has been shown in doxorubicin disposition of breast cancer (Xie et al., 2020), but there has been limited number of studies on the role of lcnRNA CBR3-AS1 (21q.22.2) in breast cancer (Zhang et al., 2020). However, the upregulation of CBR3-AS1 lcnRNA has been identified in prostate cancer and esophageal squamous carcinoma (Jiang et al., 2020). Upregulation of LncRNA DLX6-AS1 in lung adenocarcinoma (LAC) tissue reduces the level of *Distal-Less Homeobox* 6 gene (*DLX6-AS1*) mRNA and encoded protein. LncRNA DLX6-AS1 expression rate is shown to be associated with LAC differentiation (Alizadeh et al., 2020).

In the present study, we tried to investigate the probable expression change of the candidate lncRNAs, including DLX6-AS1, ZEB2-AS1, RAB6C-AS1, and CBR3-AS1, in breast cancer cells.

## Materials and Methods

*Clinical samples*

Fresh tissues were obtained from operating rooms of Shariati, Khatamolanbia, and Milad Hospitals in Tehran, Iran, by a skillful surgeon. The tissues were collected from female and one male participants with breast cancer and scoring was reported based on World Health Organization (WHO) criteria for histopathological parameters (Harbeck et al., 2019). A total of 45 tumor samples and 45 non-tumor samples (from the margin of tumor) were obtained from the same patients. Inclusion criteria included patients higher than 18 years old, signing informed consent, and not having other tumors. The Ethics Committee of Iran University of Medical Sciences, Tehran, Iran, approved the study (code: IR.IUMS.REC.1398.634).

*RNA extraction, and complimentary DNA synthesis*

Fresh samples were transferred to the RNase/DNase-free microtubes which contained RNA Later (Behnogene Co., Tehran, Iran) and were kept at -80°C. The total RNA was purified via RNA extraction kit (RNeasy, Qiagen, Inc., Chatsworth, CA) according to the manufacturers protocol. The amount of purified RNA and its quality were assessed using a NanoDrop spectrophotometer (Thermo Fisher Scientific, Waltham, USA). Also, the possible genomic DNA was removed using RNase-free DNase treatment.

A cDNA Synthesis Kit (TaKaRa Bio, Siga, Japan) was utilized for complementary DNA (cDNA) synthesis according to the protocol which uses random hexamer primers; 2µg of the total RNAs was used for this step based on the kit’s manual. The samples were incubated for 25 min at 50°C for RT enzyme activity and then incubated for 5 min at 95°C to inactivate the reaction.

*Polymerase chain reaction*

The expressions of four LncRNAs (RAB6C-AS1, DLX6-AS1, ZEB2-AS1, and CBR3-AS1) were evaluated in the present study. In order to determine the genes expressions, specific primers were designed for each LncRNA using AlleleID software (Table 1).

A gradient Polymerase Chain Reaction (PCR) was used to find the best heating and component protocol via Bio-Rad (Bio-Rad, Hercules, Calif.) instrument. The denaturation and extension steps were carried on 95°c for 30sec and 72°c for 20sec, respectively. As for amplification, 45 cycle replicates were used. A total volume of 15 µL reaction mixture, including 0.2-0.5 µM concentration of each cDNAs as template, 1× Amplicon PCR MasterMix (Amplicon Co., Denmark), 0.5 mM primer concentration (20 pmol/µl), and sterilized deionized water, was added to the total volume. Agarose gel electrophoresis system was performed to visualize the PCR products.

*Real time PCR*

Real-time PCR (RT-PCR) was carried out using 0.2-0.5 µM concentration of cDNA products or controls, LncRNA-specific primers, and 1×EvaGreen dye (Biotium, Hayward, USA). An internal control (*β2m* gene) was used to evaluate protocol efficiency. Corbett Rotorgene 3000 Real-time PCR (Corbett Research, Sydney, Australia) was implemented in this step. The first denaturation was carried out for 10 min at 95°c. The 45 amplification cycles were used as 94°C/30sec, 62°C/20sec, and 72°C/20sec. Absorbance was set at the end of extension step. Duplicate manner was performed for RT-PCR amplification and the mean was reported. In addition, a negative control was used in each run as NTC (none template control).

*Standardization and interpretation*

To standardize the protocol and gene expression control, MCF-7 cell line as a breast cancer cell line was used for RNA extraction followed by cDNA synthesis and DNase treatment. The β2M was also used as an internal control before testing and for extraction step quality control. Melt curve was analyzed in each Real-time PCR round to determine products specificity.

*Immunohistochemical Analysis*

Each block underwent staining via H&E dye and then specific antibodies were used for IHC analyses using estrogen receptor (ER) (clones 1D5 and ER-2-123, Dako), progesterone receptor (PR) (clone PgR 1294, Dako), and HER2 (HercepTest™, Dako Denmark A/S, Glostrup, Denmark) according to manufacturer’s instructions. Allred scoring system was performed for ER and PR evaluation (Harvey et al., 1999). HER2 was positive if more than 30 percent of tumor celled had higher dye in staining (+3) (Wolff et al., 2007). Each molecular subtype was determined making use of standard descriptions (Tang et al., 2009). Statistical analyses of anatomic and demographic variables were calculated in SPSS, version 16. P-values <0.05 were considered as statistically significant. 

## Results

*Patients*

Participants included 45 cases (44 females and 1 male) with the mean age ± SD of 50 ± 12.0 (range: 23-74 years). A majority of the participants had ductal carcinoma (no= 41) with 2/3 grades. IHC results showed majority of patients were scored +1 for estrogen receptor (ER) (n=28), +1 for progesterone receptor (PR) (n=26) and +1 for HER2 (n=19) but they were not significant statistically (p-value > 0.05). Table 2 shows participants’ demographic characteristics. 

*Real time PCR*

Real time PCR was used for all genes using their specific primers (Table 1). Relative expression method was used for each gene and standard curve was drawn to find the efficiency of method. Efficiency of each primer sets calculated using standard curve analysis. In this regards, primers efficiency mean calculated using standard curve analysis for B2M was 1.852, for RAB6C-AS1 was 1.798, for DLX6-AS1 was 1.865, for ZEB2-AS1 was 1.900 and for CBR3-AS1 was 1.878.

**Figure 1:**
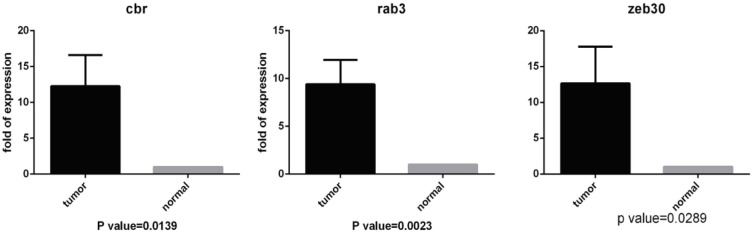
Real Time Result Analysis for Studying *CBR3-AS1, RAB6C-AS1, *and *ZEB2-AS1* Expression in the Tumor and Healthy Cell

Our results revealed no expression for *DLX6-AS1 *gene (data is not included here); however, a significant expression was observed for CBR3-AS1, RAB6C-AS1, and ZEB2-AS1 in comparison with the healthy cells (Figure 1).

*ROC curve*

**Figure 2:**
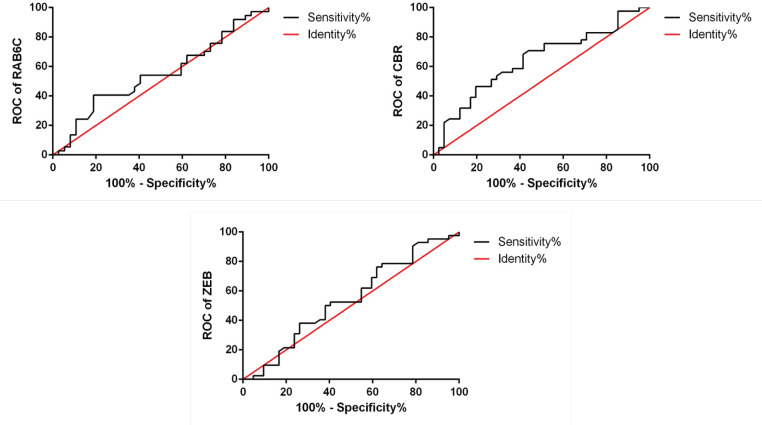
The ROC Analysis for RAB6C-AS1, CBR3-AS1, and ZEB2-AS1

ROC test was used to determine the specificity of CBR3-AS1, RAB6C-AS1, and ZEB2-AS1 expressions in the breast cancer cell. As shown in Figure 2, CBR3-AS1 LncRNA can be chosen as a breast cancer biomarker by the sensitivity more than 70%.

**Table 1:** Primer Sequences of Each Gene Used in the Present Study

Gene	Primer sequences (5'-3')		Product (bp)
*RAB6C-AS1*	F	CATTCAGAAGTGGAGAGTGTAGG	183
	R	GAGGATTGCGAGTCATCAGC	
*DLX6-AS1*	F	AGATGATTCCTGTATGTATGGCA	198
	R	AGTCTTCTTGATGATGGTGTCC	
*ZEB2-AS1*	F	GCAGAGCAGGAGAGAGACGA	152
	R	CATGCACACACCCTAATACACA	
*CBR3-AS1*	F	TGTGAGGGAGCGGGAGTC	130
	R	GTCTGGATGAGAAGAGGAAAGC	

**Table 2:** Demographic and Pathologic Characteristics of the Patients

Variables		Frequency	Percent	p-value
Total	No	45	100	-
Age	Mean	50	-	>0.05
	Std. Deviation	12	-	
	Minimum	23	-	
	Maximum	74	-	
Tumor type	Ductal carcinoma	41	91	0.02
	Fibro-adenoma	2	4	
	Lobular carcinoma	2	4	
Lymph node involvement	Not involved	24	53	>0.05
	involved by tumor	21	46	
Tumor grade	2	11	24	>0.05
	3	14	31	
	2-3	20	44	
IHC ER	Negative	12	26	>0.05
	1	28	62	
	2	5	11	
IHC PR	Negative	14	31	>0.05
	1	26	57	
	2	3	6	
	3	2	4	
IHC HER2	Negative	10	22	>0.05
	1	19	42	
	2	10	22	
	3	6	13	

## Discussion

In the current study, we made an attempt to investigate the expression of four lncRNAs in breast cancer. It was identified that DLX6-AS1 does not have any expression in tumor cells. On the other hand, RAB6C-AS1, ZEB2-AS1, and CBR3-AS1 were found to have high expressions in cancer cells which are significantly higher compared with those in healthy control tissues. Although DLX6-AS1 has expression in lung adenocarcinoma tissue (Wu et al., 2020) and human brain cells (Hu et al., 2020), our results did not show any expression in breast cancer cells. 

Long non-coding RNAs (LncRNAs) are included in human genome, which interfere with the regulation of many complexities of organisms and control many of the various biological processes, including the proliferation of stem cells and neurotransmitters (Roohallah et al., 2019). Nowadays, scientists are more interested in the role of LncRNAs in various aspects of cellular functions. Although thousands of lncRNAs are identified, only a small fraction of them have been characterized (Wang et al., 2019,Tu et al., 2020). Previously, the bioinformatic studies showed that DLX6-AS1 and GAPLINE LncRNAs do not have any expression in the breast cancer tumor cell (Alizadeh et al., 2020). It is notable that so far various studies have shown the important role of some lncRNAs in regulation, initiation, and progression of cancers (Tu et al., 2020). On the other hand, the important roles of lncRNAs have been shown in various aspects of cell biology such as epigenetics, modification, post-transcription regulation, and splicing (Tu et al., 2020). It is clear that defects in any of these activities can cause cancer. Due to the high incidence of breast cancer, finding a potential diagnostic, prognostic, and therapeutic biomarker is vital (Jiang et al., 2020).

ROC curve was used to identify the potential of each lncRNA as a biomarker for the diagnosis of breast cancer. Based on our study, CBR3-AS1 lncRNA can be a proper choice to diagnose breast cancer as a biomarker. Ronnau et al., (2014) reported CBR3-AS1 as a potential biomarker for prostate cancer (Rönnau et al., 2014). Also, a recent study showed its activity in breast cancer drug sensitivity through MAPK Signal Pathway (Zhang et al., 2020). Although Zhang et al. performed Adriamycin resistance breast cancer cells, we used fresh tissue to identify the potential lncRNA biomarker. Considering our limited sample size, we found a sensitivity of more than 70% for CBR3-AS1 in breast cancer diagnosis.

In another study, RAB6C-AS1 is reported as an inhibitor for proliferation, invasion, and metastasis and acts as a tumor suppressor similar to p53 (Fohlin et al., 2020). It is claimed that low expression of RAB6C-AS1 could have better outcomes for patients undergoing endocrine therapy (tamoxifen) (Fohlin et al., 2020). They conclude that, ER+/PR tumors with low RAB6C expression could have better outcome by tamoxifen treatment versus those with high RAB6C expression. Our study showed significant higher expression of RAB6C-AS1 in tumor cells indicating the potential resistance to some drugs like tamoxifen. Nevertheless, ER/PR profile should be examined to conclude the results. 

Also, another study showed that LncRNA ZEB2-AS1 was upregulated in breast cancer specimens and cells (MDA231) and could increase MDA231 cells proliferation and metastasis in SCID mice (Zhang et al., 2019). It has been postulated that this LncRNA could act as oncogene in breast cancer (Zhang et al., 2019). Based on our findings, we confirm significant upregulation of ZEB2-AS1 in breast cancer cells compared with adjacent marginal non-tumotic cells. This is a clue for understanding the role of ZEB2-AS1 in tumorigenesis and establishment of malignancy. However, more studies should be carried out in this subject to further confirm this finding. Our study sample size as a limitation could have influenced the results, yet, as a control group, we utilized marginal tissues. Due to limitation to find the healthy women for control group and ethical issues we had just marginal tissue as a control.

In conclusion, according to our results, it has been shown that various lncRNAs may play significant roles in breast cancer. Although RAB6C-AS1, ZEB2-AS1, and CBR3-AS1 have high expressions in breast tumor cells, only CBR3-AS1 lncRNA was observed to have a high chance to be a breast cancer biomarker via ROC analysis.

## Author Contribution Statement

All authors contributed quite equally in preparing the manuscript.
